# Use of the Extended Fujita method for representing the molecular weight and molecular weight distributions of native and processed oat beta-glucans

**DOI:** 10.1038/s41598-018-29997-0

**Published:** 2018-08-07

**Authors:** Guy A. Channell, Gary G. Adams, YuDong Lu, Richard B. Gillis, Vlad Dinu, Myriam M.-L. Grundy, Balazs Bajka, Peter J. Butterworth, Peter R. Ellis, Alan Mackie, Simon Ballance, Stephen E. Harding

**Affiliations:** 1 National Centre for Macromolecular Hydrodynamics, The University of Nottingham, Sutton Bonington, LE12 5RD UK; 2 Faculty of Medicine and Health Sciences, Queens Medical Centre, University of Nottingham, Nottingham, NG7 2HA UK; 3 School of Agriculture, The Sustainable Agricultural and Food Systems Research Division, Reading, RG6 6AR UK; 4 Biopolymers Group, Department Nutritional Sciences, Kings College London, London, SE1 9NH UK; 5 School of Food Science & Nutrition, University of Leeds, Leeds, LS2 9JT UK; 6 Nofima AS, Norwegian Institute of Food, Fisheries and Aquaculture Research, Ås, Norway; 7 Kulturhistorisk Museum, Universitetet i Oslo, Postboks 6762, St. Olavs plass, 0130 Oslo, Norway

## Abstract

Beta 1–3, 1–4 glucans (“beta-glucans”) are one of the key components of the cell wall of cereals, complementing the main structural component cellulose. Beta-glucans are also an important source of soluble fibre in foods containing oats with claims of other beneficial nutritional properties such as plasma cholesterol lowering in humans. Key to the function of beta-glucans is their molecular weight and because of their high polydispersity - molecular weight distribution. Analytical ultracentrifugation provides a matrix-free approach (not requiring separation columns or media) to polymer molecular weight distribution determination. The sedimentation coefficient distribution is converted to a molecular weight distribution via a power law relation using an established procedure known as the Extended Fujita approach. We establish and apply the power law relation and Extended Fujita method for the first time to a series of native and processed oat beta-glucans. The application of this approach to beta-glucans from other sources is considered.

## Introduction

**Figure 1:**
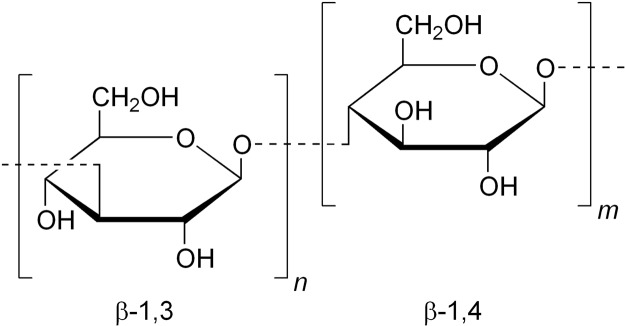
Part of the 1–3, 1–4 β-D-glucan molecule. https://commons.wikimedia.org/wiki/File:Beta-1,3-1,4-glucan.png.

Beta 1–3, 1–4 glucans, “beta-glucans”, are one of the key polysaccharides of the cell walls of oats and other cereals, complementing the main structural component cellulose^1–3^. Beta-glucans are also an important source of water soluble dietary fibre in oat-containing foods with claims of other beneficial nutritional properties such as promoting plasma cholesterol reduction and lowering postprandial glycaemia^4,5^. Besides their chemical structure (Fig. 1), the key to the function of beta-glucans in the plant, and their use (or potential use) commercially or in biomedicine is their molecular weight and, because of their high polydispersity – their molecular weight distribution. In some cases, preparations of beta-glucan can be readily soluble and have fairly unimodal distributions of size. In other cases, insoluble components or impurities are present^6^ and unless removed can cause problems with several techniques used to characterise the physical properties of the soluble components. A recent study^7^ has considered solubility and dissolution kinetics and linked this to the biological activity of beta-glucan.

An approach for polydisperse polymers was recently published^8^ based on the matrix-free technique of sedimentation velocity in the analytical ultracentrifuge and converts a distribution of sedimentation coefficient g(*s*) vs *s* plot into a distribution of molecular weight utilising the power-law or scaling relationship between the sedimentation coefficient and molecular weight^9–11^:

1a $$s={{\rm{\kappa }}}_{{\rm{s}}}{M}_{w}^{b}$$

where *s* is the sedimentation coefficient, *M*_w_ is the weight average molecular weight and *κ*_s_ and *b* are characteristic coefficients related to conformation. For example, *b* = 0.4–0.5 for a coil type of conformation, ~0.15–0.2 for a rod conformation and ~0.67 for a spherical conformation.

The sedimentation coefficient *s* depends on the size and shape of the macromolecule^12^ but if *κ*_s_ and *b* are known then *M*_w_ can be found from:

1b $${M}_{w}={(s/{{\rm{\kappa }}}_{{\rm{s}}})}^{1/b}$$

The method has been applied to alginate, pectins, and mucin glycoproteins from a wide variety of sources^13^. We establish and apply the power law relation for the first time for beta-glucans based on work on oat beta-glucan BG90. Then for a series of beta-glucans of lower degree of purity we illustrate the simple application of the scaling equation, Eq. (1b) to identify the weight average molecular weights from the sedimentation coefficients. Finally, we use the Extended Fujita approach^8^ to evaluate the molecular weight distributions of beta-glucans extracted from oats that had been processed by roller milling and subject to long-time storage for 3 years at 21 °C.

A major development in the molecular weight determination of polysaccharides occurred 3 decades ago with the introduction of the SEC-MALS method (size exclusion chromatography coupled to multi-angle light scattering)^14–16^, which provides separation and absolute molecular weight analysis. The first application of this method to polysaccharides – namely alginates - was in 1991^11,17^, and to glycoconjugates in 1996^18^, and is a technique which has now become the chosen method for many polymer systems.

However, there are two major limitations which restricts the types of molecule that can be successfully analysed, (i) the separation limit for the columns (with generally an upper limit of ~2–3 × 10^6^ g/mol), and (ii) lack of inertness of the columns used. Some beta-glucans^5^ have very large molecular weights (either native or through aggregation phenomena) that exceed the molecular weight limit for separation on the columns, and many offer solubility problems that can affect the inertness of the columns. Issues of solubility and non-unimodality can also affect other methods such as sedimentation equilibrium in the analytical ultracentrifuge and our recently developed SEDFIT-MSTAR method^19^. The Extended Fujita approach based on sedimentation velocity at high speeds is more suited and supra-molecular impurities are automatically removed. The approach offers a complementary alternative to SEC-MALS and sedimentation equilibrium and can be applied to situations in which other methods cannot be employed.

### The Extended Fujita approach of Harding, Schuck & coworkers

Fujita^20^ provided the basis for converting a (differential) distribution g(*s*) of the sedimentation coefficient *s* into a (differential) distribution f(*M*) of the molecular weight M for linear polymers, based on the assumption that the polymers behave as randomly coiled polymers in solution, with *b* = 0.5 in Eq. (1). Harding, Schuck and colleagues^8^ provided a generalisation of this method to cover any conformation type (including spheres, rods and coils). g(*s*) is defined as the weight fraction of the species with a sedimentation coefficient between *s* and *s* + d*s* and f(*M*) is defined as the weight fraction of species with a molecular weight between *M* and *M* + d*M*. To transform g(*s*) vs. *s* to f(*M*) vs *M* firstly:

2 $${\rm{g}}(s).ds={\rm{f}}(M).dM$$

and so

3 $${\rm{f}}(M)={\rm{g}}(s)(ds/dM)$$

where

4 $$ds/dM=b.{{{\rm{\kappa }}}_{{\rm{s}}}}^{1/b}.{s}^{(b-1)/b}$$

Therefore, to perform the transformation the conformation type or *b* needs to be known under the particular solvent conditions and at least one pair of *s-M* values is needed to define the *κ*_s_ from Eq. (4). Care needs to be expressed concerning thermodynamic/hydrodynamic non-ideality^21^, but these effects can be avoided by working at low polymer concentrations, taking advantage of the fact that sedimentation velocity experiments can be performed at concentrations as low as 0.1–0.2 mg/ml, where such effects are usually negligible. The method has now been built into the popular sedimentation velocity analysis platform known as SEDFIT^22,23^. In the transformation from g(*s*) vs *s* to f(*M*) vs *M* a diffusion corrected variant of g(*s*) known as c(*s*) is sometimes used^22,23^.

### Evaluating the scaling parameters *κ*_s_ and *b* for oat beta glucans

**Figure 2:**
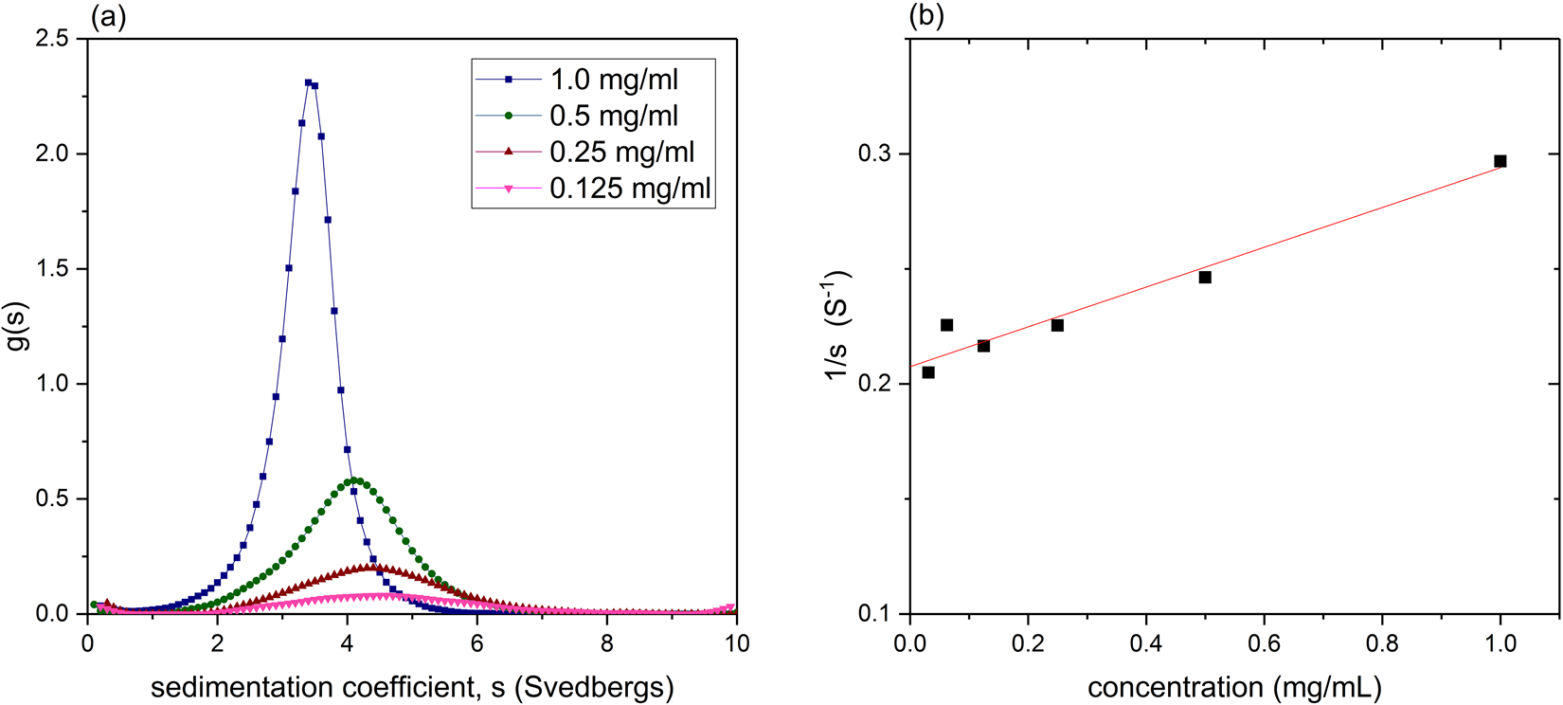
Analytical ultracentrifugation of oat beta-glucan BG90. (**a**) Sedimentation coefficient distribution plots g(*s*) vs *s* in phosphate-chloride buffer pH = 6.8, I = 0.10 M, at 3 serial dilutions from 1.0 mg/ml. A rotor speed of 40000 rpm was used. (**b**) Reciprocal plot of *s* versus concentration, fitted to (1/*s*) = (1/*s*°).(1 + *k*_s_*c*) where *k*_*s*_ is the concentration dependence or ‘Gralén’ coefficient (Gralén, 1944; Harding & Johnson, 1985). From the fit a value of *s*° = (4.82 ± 0.10)S and *k*_s_ = (420 ± 40) ml/g are obtained.

We can now seek to develop the procedure for oat beta-glucans by establishing the *κ*_s_ and *b* parameters. To do that, we select a beta-glucan, which is fully soluble and has a unimodal distribution of sedimentation coefficient. One such material is a substance known as oat beta-glucan BG90, supplied by F. Prothon (Swedish Oat Fibre, Bua, Sweden) that has previously been well characterised chemically (see ref.^7^, where it is referred to as “BG2”). Its g(*s*) vs *s* distribution for a series of concentrations (where *s* is the apparent sedimentation coefficient, i.e. not corrected for non-ideality, at concentration *c*) is shown in Fig. 2a. We then obtain the *b* and *κ*_s_ in the following way:

#### Defining *b*

**Figure 3:**
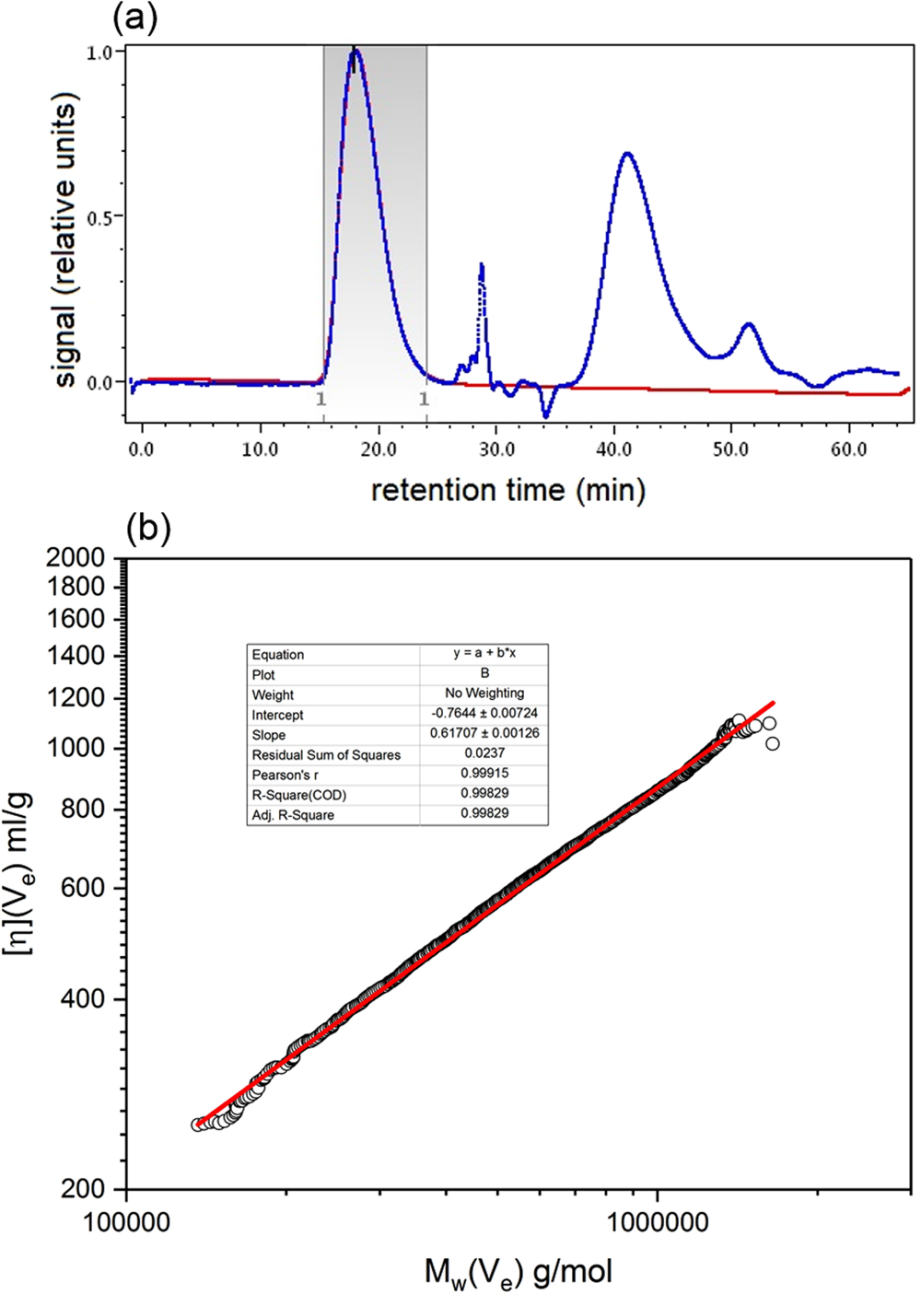
SEC-MALS of oat beta-glucan BG90. (**a**) Elution profile with the beta-glucan peak limits selected in grey. Blue line: refractrometric (concentration) signal. Red line (light scattering signal recorded at a scattering angle of 90°). Both profiles normalized to a maximum of 1.0. (**b**) Mark Houwink-Kuhn Sakurada (MHKS) plot of intrinsic viscosity [η](*V*_e_) values versus molecular weight *M*_w_(*V*_e_)  corresponding to elution volume values *V*_e_ within the marked limits of (**a**). Fit parameters shown in the inset.

We use SEC-MALS coupled to an on-line viscometer. Figure 3a shows the SEC elution concentration profile recorded using the on-line differential refractometer and one of the light scattering detectors. We then record, as a function of elution volume *V*_e_, the intrinsic viscosity [η](*V*_e_) versus weight average molecular weight *M*_w_(*V*_e_) profile and from the slope of a plot of log-log plot (Fig. 3b) across the main peak of elution volumes (*V*_e_) evaluate the viscosity power law (scaling) coefficient *a*. It should also be remarked that across the selected peak the very accurate linear fit is indicative of a pure component within this range. From Fig. 3b a value of *a = *0.62 corresponds to a flexible coil conformation. We can then use the Tsvetkov relation^9^ linking the sedimentation and viscosity power law coefficients to obtain a value of 0.45 for *b*:

5 $$b=(2-a)/3$$

We then repeated the whole procedure on a different SEC-MALS instrument in a different institution and re-assuringly obtained a similar value *b* = 0.45. This gave us a working value for *b* = (0.455 ± 0.010).

#### Defining *κ*_s_

To do this, we simply use Eq. (1) with the weight average sedimentation coefficient *s*, the weight average *M*_w_ from SEC-MALS and a *b* value of (0.455 ± 0.010). The weight average sedimentation coefficient after extrapolation to zero concentration (Fig. 2b) to remove non-ideality effects was *s*° = (4.82 ± 0.10)S. The values for *M*_w_ from both instruments were 634,000 and 651,000 g/mol so we take *M*_w_ = (642,000 ± 10,000) g/mol, and because of the very low concentrations after dilution on the columns, non-ideality effects can be ignored. This gives a value for κ_s_ under high dilution conditions (*c* < 0.15 mg/ml):

6 $${{\rm{\kappa }}}_{s}=(s/{M}_{w}^{b})=(4.82/{64000}^{0.455})=0.01098$$

**Figure 4:**
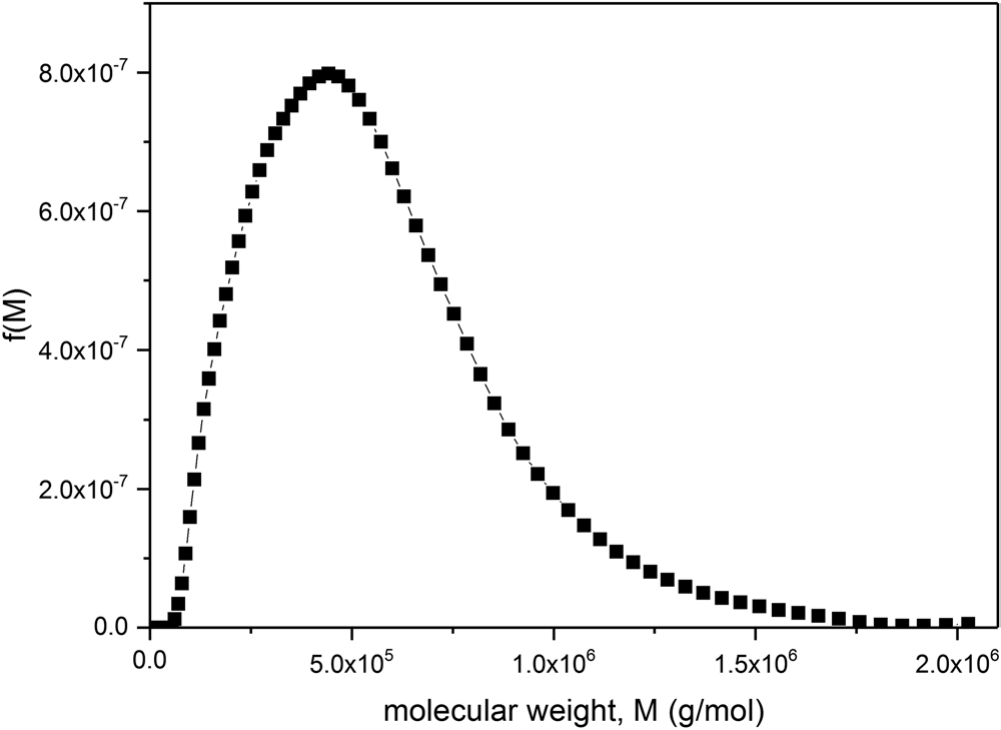
Molecular weight distribution f(*M*) vs *M* for oat beta-glucan BG90. After transformation from the g(*s*) vs *s* distribution for *c* = 0.125 mg/ml (Fig. 2a), with coefficients *b* = 0.455 and *κ*_s_ = 0.01908.

Figure 4 shows the distributions obtained in this way for oat beta-glucan BG90. One can see the broad distribution and the large amount of high molecular weight material >500,000 g/mol.

### Exploring the concentration limit for transformation of a g(*s*) vs *s* plot to an f(*M*) vs *M* plot

Where distributions g(*s*) vs *s* cannot be obtained under high dilution conditions (i.e. where *s* ~ *s*°) an approximation can be made that the weight average *s* value at that concentration is substituted into Eq. (6) and the appropriate *κ*_s_ value selected (Table 1). The appropriate sedimentation coefficient value, *s*, for that concentration, *c*, can be found using the Gralén relation^24^:

7 $$(1/s)=(1/{s}^{o}).(1+{k}_{s}c)$$

where *k*_s_ is the concentration dependence or ‘Gralén’ coefficient (see refs^21,24,25^), where (Fig. 2b) *k*_s_ = (420 ± 40) ml/g. Note that the Gralén coefficient should not be confused with the power-law scaling coefficient *κ*_s_.

**Table 1:** Scaling *b*, κ_s_ values for transforming sedimentation coefficient distributions for oat beta-glucans.

concentration	*b*	*κ*_s_
<0.15 mg/ml	0.455	0.01098
0.2 mg/ml	0.455	0.01037
1 mg/ml	0.455	0.00768

**Figure 5:**
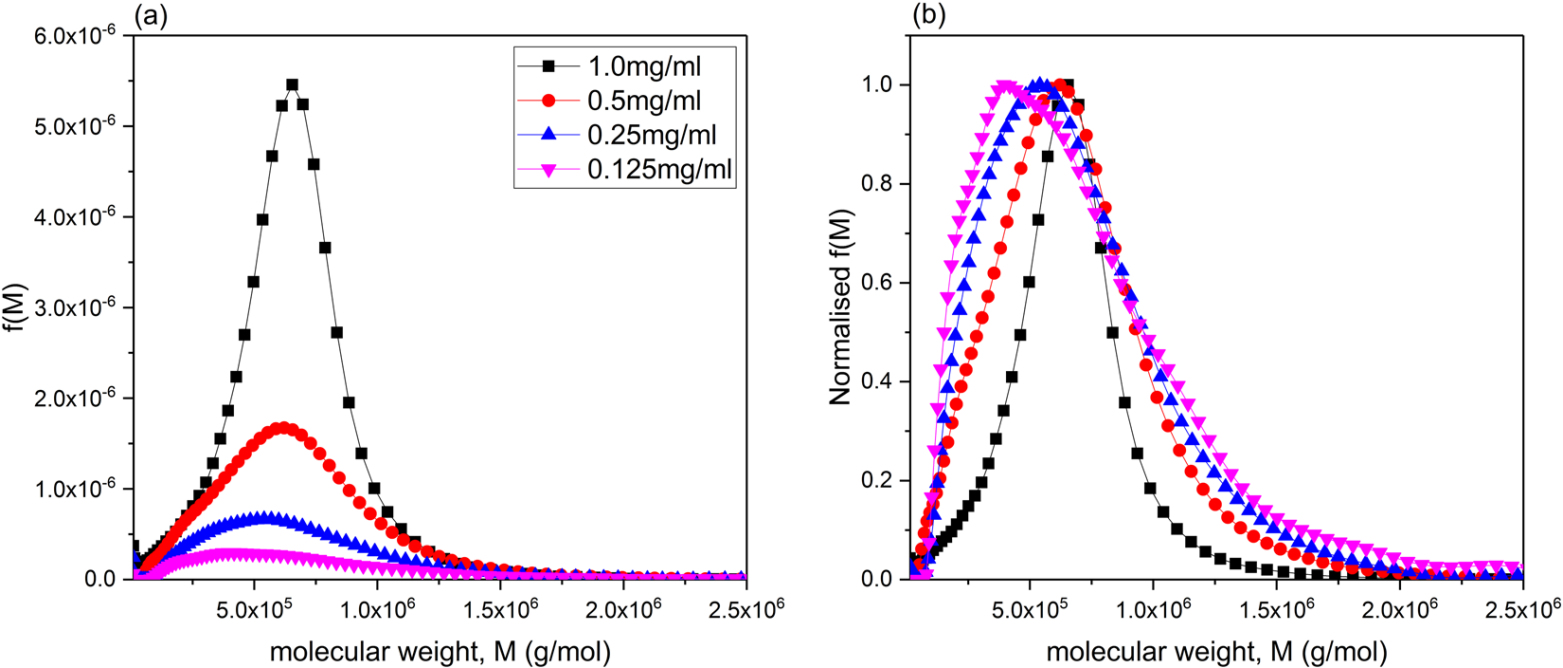
Apparent molecular weight distributions for oat beta-glucan BG90. (**a**) obtained at 0.125 mg/ml, 0.25 mg/ml, 0.5 mg/ml and 1 mg/ml; (**b**) as (**a**) but normalized so the maximum value for f(*M*) = 1.0.

Figure 5a shows the corresponding estimated apparent molecular weight distributions of BG90 beta-glucan for different concentrations. It is seen that at 1.0 mg/ml significant hypersharpening effects due to the combined effects of polydispersity and non-ideality are evident but for concentrations *c* < 0.5 mg/ml distortions of the distribution are not significant and the approximation appears to be reasonable, as is evident from the normalised distribution plots of Fig. 5(b). We recommend working at low concentrations unless specific concentration effects such as possible aggregation phenomena are being explored.

### Application to beta-glucans from oats processed by roller milling and long-term storage

**Figure 6:**
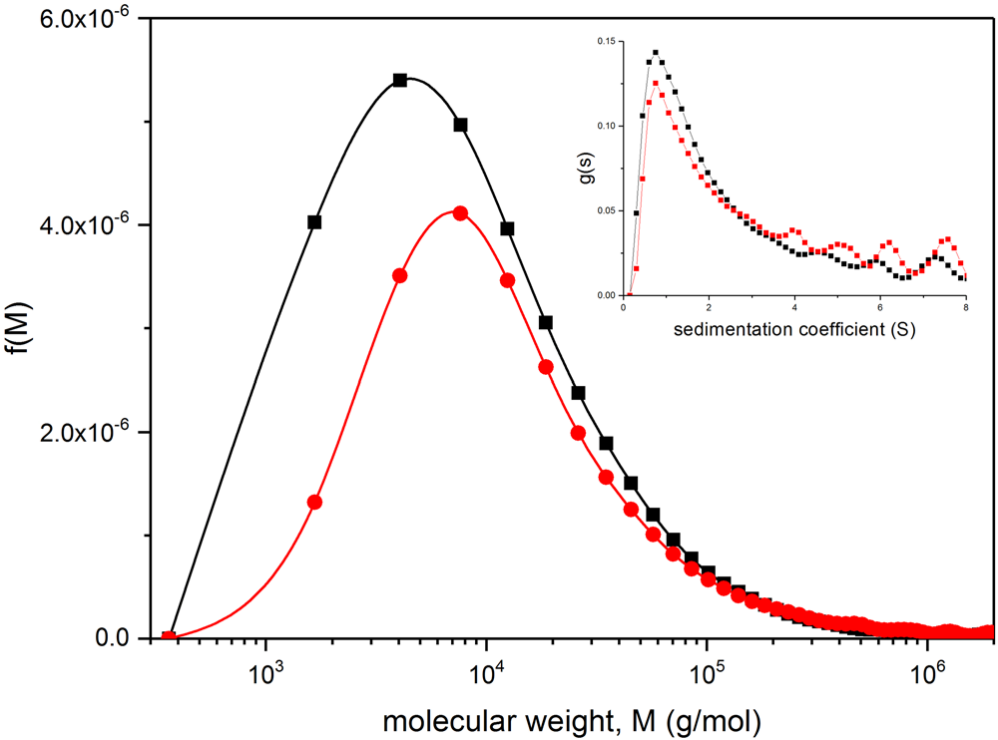
Molecular weight distribution f(*M*) vs *M* for beta-glucans in two roller milled and stored (3 years at 21 °C) oat samples (Matilda variety). Loading concentration *c* = 0.2 mg/ml. *b* = 0.455, κ_s_ = 0.01037. Inset: corresponding sedimentation coefficient distribution. Black line and squares: 200 μm aperture sieve used in the processing. Red line and circles: 710 μm.

To further illustrate application of the method we looked at two preparations of beta glucans extracted from rolled oats and stored for a long period (>3 yrs at 21 °C); oat flakes and flour were prepared from the Matilda variety (for details see Methods section). The sedimentation coefficient distributions were recorded at the low concentration of 0.2 mg/ml. The value of *κ*_s_ is adjusted accordingly (Table 1), and the corresponding distributions shown in Fig. 6 show the broad range of sizes. The low weight average molecular weights observed in these samples may be due to residual beta-glucanase activity and prolonged storage although the distribution follows a similar log-normal form as the BG90 purified beta-glucan. This would also account for the lower molecular weight in the 200 μm vs 710 μm samples due to the increase in accessibility of the beta-glucan to the beta-glucanase. In addition, it is possible that small quantities of mainly solubilised amylose, arising from leaching of swollen, gelatinised starch granules in the original oat flour, may have contributed to the molecular weight distribution profiles.

### Simple power-law application

If simply the (whole distribution) weight average molecular weights *M*_w_ rather than the distributions of f(*M*) vs *M* are sought then these can be obtained directly from the sedimentation coefficient from Eq. (1b). Table 2 ^6,26–28^ shows values of some other oat beta glucans obtained in this way, again at a loading concentration of 0.2 mg/ml.

**Table 2:** Sedimentation coefficients of oat beta-glucans from the Oatwell 32 series (0.2 mg/ml), and corresponding molecular weights^a^.

Sample	*s* (S)	10^−6^ × *M*_w_(g/mol)
Oat 32 extract, Rieder *et al*.^26^ with Na_2_SO_4_	9.24	3.05
Oat 32 extract minus Na_2_SO_4_ + 2^nd^ peak^b^	8.68 22.5	2.70 22.8
Oat 32 extract, Beer *et al*.^27,28^	8.63	2.62
Oat 32 extract, Wang *et al*.^6^	6.61	1.46

^a^Evaluated from Eq. (1b). ^b^This may represent part of the supra-molecular aggregate or non-beta-glucan material.

## Discussion

The inherent fractionation ability of the sedimentation velocity method without the need for columns or membranes, together with the automatic removal of supramolecular impurities appears to be useful for impure or incompletely soluble materials. The Extended Fujita method has been incorporated into the SEDFIT suite of analytical ultracentrifuge algorithms, developed by Schuck & coworkers (see Harding *et al*. 2011^8^, Brown & Schuck^23^) and is readily useable. The method is seen as complementary to SEC (or FFF) –MALS, which have been established as the method of choice for many polymeric molecular weight distribution analyses, and, for polysaccharides since the first analyses in 1991^17^. However, the latter methods are not useful in cases where non-inertness of the columns (SEC) or membranes (FFF) are suspected, there is poor solubility or the separation range has been exceeded.

The Calcofluor fluorescence-labelled chromatography method^7,29^ – specific for β(1–4) bonds - has also proved useful for the analysis of the molecular weight of beta-glucans in the presence of non β(1–4) containing impurities, although for distributions containing substantial amounts of high molar mass material >500,000 g/mol the Calcofluor method results in an increasing underestimation of the molecular weight^7,29^. For such systems the Extended Fujita method also provides a useful complementary approach. We are now exploring the extension of our approach to other beta-glucans, most notably from barley and wheat.

## Methods

### Beta-glucans

Oat beta-glucan BG90 was solubilised according to the following method: 0.0280 g of Oat BG90 β-glucan was accurately weighed into the base of a pre-weighed 50 ml dry pyrex conical flask and 5 ml of 0.1 M pH 7 PBS (I = 0.1 M) buffer was added to pre-wet the sample. A further 15 ml aliquot of buffer was then added ensuring all solid material was washed below the liquid surface. A pre-weighed magnetic stirrer bar was added with a pre-weighed ‘3-port PTFE Duran bottle insert’ (port 1- thermocouple wire, port 2-sealed injection, port3 –blank), designed to prevent evaporative loss. The mixture was then placed on a hot-plate magnetic stirrer at 80.0 °C for 1 hr. A final 5 ml aliquot of buffer was added again ensuring any solid material was washed below the liquid surface, and heated for a further 1 hr at 80.0 °C until dissolved. Oat BG90 solution was then extensively dialysed (>24 hr, 1 change) against the 0.10 M pH 7.0 phosphate-chloride buffer^30^. It was then made up to a stock concentration, *c*, of 1.0 mg/ml (measured refractometrically). No significant protein contaminant was evident (from the absence of uv-absorption at a wavelength of 280 nm).

Oat flakes from the Matilda variety were obtained from Lantmännen Cerealia, Moss, Norway. Oat flour was produced at Campden BRI (UK) by milling the same oat flakes on a laboratory hammer mill (Christy, 8 Lab Mill, Christy & Norris Limited, Chelmsford, UK) fitted with either a 5.0 or 0.25 mm sieve. Two different particle size fractions were obtained using two hand sieves of 200 and 710 μm aperture. The oat flours were hydrated in PBS buffer to solubilise the beta-glucan as with BG90. Again, from the absence of uv-absorption at 280 nm, no significant protein contaminant was evident.

### Analytical Ultracentrifugation

Sedimentation coefficient distributions were evaluated using the Beckman Optima XL-I analytical ultracentrifuge (Beckman Instruments, Indianapolis, USA). A volume of 400 µl of beta-glucan solution and matching amounts of buffer were injected into appropriate channels of 12 mm double sector aluminium epoxy cells with sapphire windows. Solutions were centrifuged at 40000 rpm at a temperature of (20.0 ± 0.1)°C. The weight average sedimentation coefficient ‘*s*’ (in Svedbergs, S) for a particular component was then corrected to standard solvent conditions (the density and viscosity of water at a temperature of 20.0 °C). A partial specific volume of 0.61 ml/g was used^31^.

### SEC-MALS

The SEC consisted of a Postnova Analysis PN7505 degassing unit (Landsberg am Lech Germany), Shimadzu LC-10AD HPLC Pump (Shimadzu UK, Milton Keynes, UK.), fitted with a Spark-Holland Marathon Basic autosampler (Spark Holland, Emmen, The Netherlands) combined with a TSK Gel guard column (7.5 × 75 mm) and TSK Gel G5000, G6000 columns (7.5 × 300 mm) connected in series (Tosoh Biosciences, Tokyo, Japan). Light scattering intensity was detected using a DAWN^®^ HELEOS™ II, light scattering photometer connected in series to a ViscoStar^®^ II on-line differential viscometer, an Optilab^®^ rEX refractive index detector (Wyatt Technology Corporation, California, U.S.A.). The stock solution of 1.0 mg/ml was filtered through a 0.45 µm syringe filter (Whatman, Maidstone, England) - to remove any insoluble material or dust prior to injection - and then injected into the autosampler. A 100 µL aliquot of each solution was injected onto the columns at ambient temperature. The eluent employed was the PBS dialysate at a flow rate of 0.8 mL/min. ASTRA^™^ (Version 6) software (Wyatt Technology Corporation, Santa Barbara, U.S.A.) was used to estimate the weight average *M*_w_ and z-average *M*_z_ molecular weights with a 1^st^ order Zimm extrapolation^13,14^. Because of the low solute concentrations after dilution on the columns non-ideality effects were assumed as negligible. A refractive increment (*dn/dc*) ~0.146 mL/g was used^32^.
